# Neighborhood built environment and fracture risk among older adults: comparing high versus low clusters

**DOI:** 10.3389/fpubh.2026.1947343

**Published:** 2026-10-16

**Authors:** Qiaohui Zhou, Riken Homma

**Affiliations:** 1 Culture and Tourism College, Jinhua University of Vocational Technology, Zhejiang, China; 2 Faculty of Advanced Science and Technology, Kumamoto University, Kumamoto, Japan

**Keywords:** bone fracture, built environment, cluster, older adults, walkability

## Abstract

Osteoporotic fracture is a leading cause of disability and a major contributor to medical care costs worldwide. Given that over half of fractures occur outdoors, understanding the relationship between the outdoor environment and fracture risk is essential. However, few studies have investigated the relationships between built environment and fractures. No study has explored how built environment attributes relate to fractures across different geographical areas. In this study, utilizing a five-year period of fracture data and fine-grained residential point data, we employed ordinal scan statistics to identify high and low fracture clusters among Japanese older adults. We then applied a zero-inflated ordered logit (ZIOL) model within each cluster to examine the associations between built environment attributes and fracture occurrence as well as fracture frequency. As a result, we identified one high and one low fracture clusters. In the low cluster areas, no significant associations were found between built environment attributes and fracture occurrence as well as fracture frequency. In contrast, in the high cluster areas, four individual built environment attributes—population density, intersection density, accessibility of bus services, and mix of function—showed statistically significant negative associations with fracture occurrence in their respective models. Notably, population density was particularly significant. This research provides valuable insights for developing preventions aimed at promoting active and independent aging by targeting specific neighborhood characteristics.

## 1 Introduction

Osteoporotic fracture is one of the most common causes of disability and a major contributor to medical care costs globally (1). In 2010, it was estimated that 21 million men (3.1%) and 137 million women (18.2%) had a high probability of osteoporotic fracture worldwide. This number is projected to almost double, reaching a total of 319 million by 2040 (2). The greatest number of individuals at high risk were from Asia, accounting for 55% of the total. Another study also pointed out that by 2050, more than 50% of all osteoporotic fractures will occur in Asia due to the significant aging of the population in this region (3). Japan is particularly notable for its high aging population (aged 65 and above). According to the Japanese Ministry of Health, Labor and Welfare, the costs related to deficiencies of bone density and bone structure, as well as fracture-related expenditure for aging population, were estimated to be almost JPY 1,278 billion in 2021 (4). Meanwhile, the aging population in Japan is projected to increase to 37.7% by 2050 (5). This demographic shift will undoubtedly lead to heavier societal and economic burdens (6).

Neighborhood built environment plays an important role in facilitating physical activity behavior and affecting health outcomes. This is particularly true for older adults, as they are more dependent on their proximal and local neighborhood environment due to declines in physical and cognitive functions (7). The World Health Organization’s Global Age-friendly Cities guide outlines key neighborhood domains that promote active aging and support aging in place through combined natural, built, and social environments. Built environment is often conceptualized in terms of walkability (8). Previous study suggested that over half of fractures occurred outdoors, making it crucial to analyze the outdoor environment and fracture risk (9). However, few studies have investigated the relationship between walkability and fractures. Zhu et al. found that long-term exposure to walkability, measured using a modified walk score tool, was associated with a reduced incidence of fractures (10). Similarly, Wende identified a significant relationship between walkability and total fractures among postmenopausal women (8). A study in Japan suggested that easier access to facilities and bus services were associated with reduced fracture risk among older people (11). Despite these important findings, there remains a significant gap in understanding on how built environment attributes correlate with fracture risk in spatially concentrated high risk areas. As Yiannakoulias et al. emphasized, a fundamental element of health outcomes research is to quantify spatial variations in disease incidence and examine how these variations correspond to underlying spatial risk factors (12). Geographical approaches play a critical role in providing exploratory insights into the spatial distribution of health outcomes, enabling researchers to identify high risk areas and develop more targeted, evidence-based intervention strategies. Macintyre et al. further highlighted that analyzing geographic variations in health outcomes serves as an essential first step in making more informed, practical, and immediate decisions regarding disease prevention and intervention efforts (13). Therefore, identifying fracture hotspots and analyzing the specific built environment factors associated with fracture risk within these clusters can provide key insights for designing location-specific prevention strategies.

To address these gaps, we utilized five years of fracture records and fine-grained residential point data to identify fracture clusters among older adults. We then examined associations between each built environment indicator and fracture outcomes within the identified high and low clusters separately. We hypothesize that built environment attributes, particularly walkable environments, are associated with a reduced risk of osteoporotic fractures among older adults. Walkable environments encourage physical activity, which plays a crucial role in maintaining bone density, muscle strength, and balance, and ultimately enhance fall prevention and reduce fracture risk (14). Furthermore, we hypothesize that the relationship between walkability and fracture risk may vary between high and low cluster areas due to differences in population characteristics and environmental conditions. In high cluster areas, walkable environments may have a more significant association since these areas already exhibit a high concentration of fractures, improvements in walkability can play a crucial role in promoting physical activity, preventing falls, and ultimately mitigating fracture risk. Conversely, in the low cluster areas, the association between walkability and fracture risk may be less pronounced. While walkable environments may still provide health benefits, the lower baseline fracture incidence suggests that other factors may play a more dominant role in determining fracture risk. This research provides exploratory evidence on spatial clustering of fracture outcomes and on the observed patterns of association between built environment characteristics and fracture outcomes across high and low fracture cluster areas. The findings will help to pinpoint areas requiring urgent intervention and provide a basis for developing hypotheses for causality analysis.

## 2 Materials and methods

### 2.1 Data

We utilized two types of data: health data and built environment data. Health data were obtained from the Kumamoto Prefecture Senior Citizens’ Medical and Wide Area Alliance. The dataset includes fracture data of 39,577 older adults aged 75 or above over the period from 2017 to 2021. Each older adult was assessed annually, with fracture occurrence recorded as either yes or no for each year, resulting in an ordered variable ranging from 0 to 5, categorized into six categories: 0 (no fracture), 1, 2, 3, 4, and 5. This variable represents fracture frequency, that is, the number of years in which a fracture was recorded, rather than clinical fracture severity; we therefore use the term fracture frequency hereafter. In addition to fracture data, the health data also encompassed detailed information on various characteristics, such as age, gender, physical examination results, laboratory test results, medical history, and medication use.

Regarding the built environment, we employed six indicators: walk score, mix of function, population density, intersection density, accessibility of bus services, and slope. The data sources and measurement methods used in this study are consistent with those described in a previous publication (11). Walk score was a numerical measure of walkability based on the proximity of a residence to 12 categories of facilities (i.e., health facility, supermarket, convenience store, bank & post office, public assembly space, park, outdoor & sports facility, drug store, grocery store, catering establishment, cultural facility, and religious facility) commonly used by older adults. The mix of function was calculated as the diversity of facilities within walking distance. A higher value indicates a more varied mix of facilities, which is associated with increased walkability as it provides greater opportunities for various activities and services. Intersection density was the number of intersections within walking distance. Higher intersection density is associated with more walkable environments, as it reduces travel distances and creates shorter routes for pedestrians. Accessibility of bus services was the ease with which older adults can access public bus transportation within walking distance. It involves considering the proximity of residence to bus stops and the frequency of bus services. Population density was defined as the number of persons at each 5th mesh (length side ≈ 250 m) where each older adult resided. Slope was captured at the location of each older adult’s residential address using surface parameter tool in ArcGIS pro. Walk score, mix of function, intersection density, and accessibility of bus services were assessed within a 500-meter network walking distance buffer zone based on the residential address coordinates (38,987 (98.51%) were geocoded at the household level and 590 (1.49%) were geocoded at the block level) of the participants, as the Japan Ministry of Land, Infrastructure, Transport and Tourism notes that walking distance for older adults is approximately 500 meters (15). By measuring health and built environment indicators at the precise individual level, we avoided issues related to the modifiable areal unit problem, resulting in more accurate and reliable results.

### 2.2 Methods

Figure 1 presents the analysis framework of the study. First, we identified the high and low clusters of fracture risk using Kulldorff’s spatial scan statistics. Then, based on the detected clusters, we employed a zero-inflated ordered logit (ZIOL) model to examine the relationships between built environment attributes and fracture outcomes within each cluster separately. The ZIOL model showed a substantially higher log likelihood and lower AIC and BIC values than the generalized ordered logit model, indicating substantially better statistical fit despite its greater number of parameters. Detailed descriptions of Kulldorff’s spatial scan statistics and the ZIOL model are provided in Sections2.2.1 and 2.2.2, respectively.

**Figure 1:**
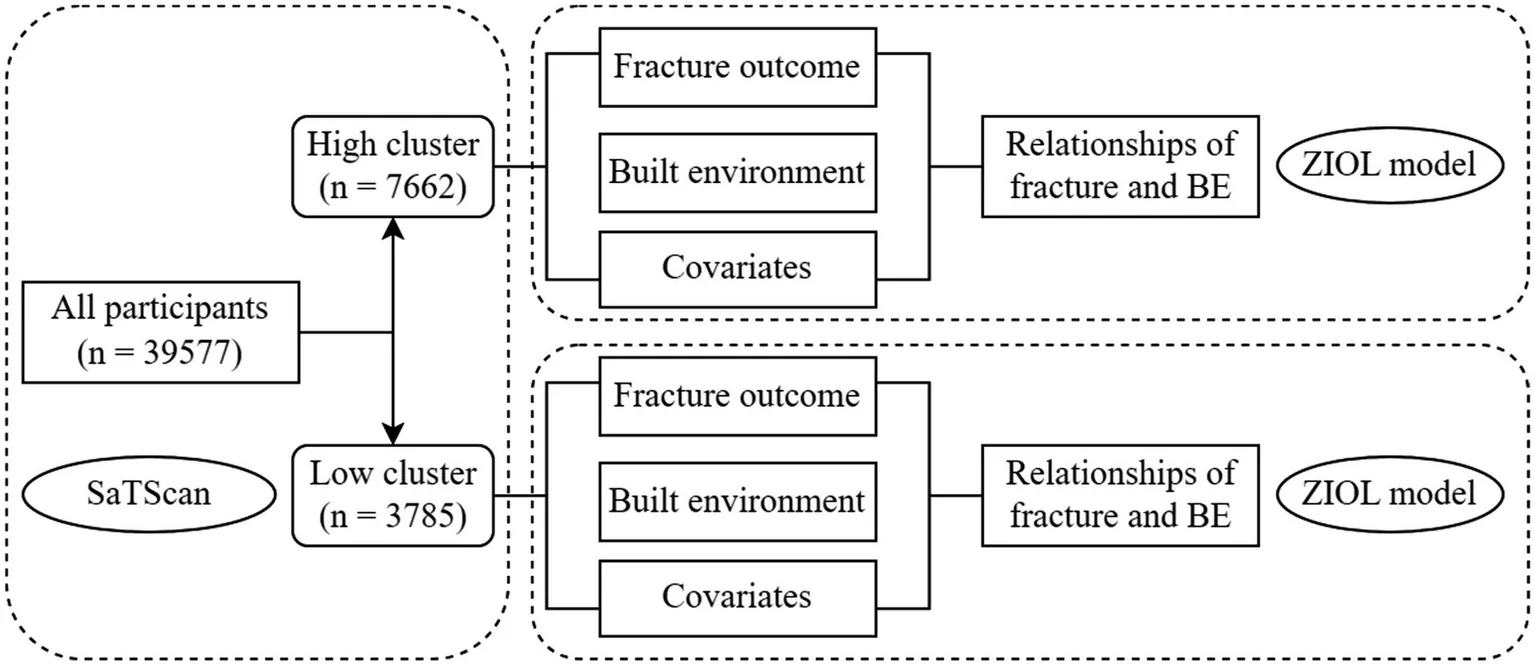
Analysis framework.

#### 2.2.1 Ordinal spatial scan statistics

Spatial clusters, or disease hotspots, are defined as an unusual numbers of cases within a population, palace and time period (16). Numerous methods have been developed to detect local clusters in point data, with Kulldorff’s spatial scan statistics being one of the most commonly utilized techniques (17). A scoping review indicated that over half of the reviewed papers applied Kulldorff’s spatial scan statistics to analyze the spatial pattern of point data (18). This technique is widely used across various fields, including epidemiology (12, 19–21), sexual risk behaviors (22), natural disasters (23), and engineering (24).

Kulldorff’s spatial scan statistics is based on the likelihood ratio test. The method identifies clusters by placing a scanning window at different spatial coordinates within the study area and identifying the most likely clusters based on the maximum value of this test statistic. Different probability models can be employed depending on the nature of the data. For ordinal data, it is preferable to use ordinal model that incorporates the ordinal structure rather than dichotomizing the data, which can result in information loss (25). The significance of the test statistic is evaluated using Monte Carlo hypothesis testing.

In this study, we used software SaTScan version 10.1 with an ordinal model to detect spatial distribution patterns of fracture risk for older adults in Kumamoto Prefecture. The maximum spatial cluster size was set at 50%, as recommended by the SaTScan User Guide for version 10.1 (26). To enhance the robustness of the scan statistics, Gini coefficients were used to optimize the maximum reported cluster size (MRCS) (27, 28). Monte Carlo simulations (n = 999) were conducted to obtain relevant *p* values and 95% confidence intervals, with statistical significance determined at the 0.05 alpha level. The output files were further processed using ArcGIS Pro for visualization and following analysis.

The ordinal spatial scan statistic was proposed by Jung et al. (25). Formally, it assumes a study area composed of I sub-regions and an outcome variable recorded in K categories. $C_{\mathrm{ik}}$ is the number of observations in location i and category k, where i = 1, …, I and k = 1, …, K. Given scanning window Z, $p_{k}$ is the unknown probability that an observation within the scanning window Z belongs to category k, while $q_{k}$ is the unknown probability that an observation outside the scanning window Z belongs to category k. The null hypothesis is that the probability of being in category k is the same as inside and outside the scanning window ( $H_{0}:p_{1}=q_{1},\ldots ,p_{K}=q_{K}$ ). The alternative hypothesis is considered as


$$H_{a}:\frac{p_{1}}{q_{1}}\leq \frac{p_{2}}{q_{2}}\leq \ldots \leq \frac{p_{K}}{q_{K}}$$

With at least one inequality being strict, this ensures that detected clusters represent an area with higher rates of high fracture occurrences compared to the surrounding area. The likelihood ratio test is expressed as


$$\lambda_{z}=\frac{\prod_{k}(\prod_{i\in z}{{\hat{p}}_{k}}^{c_{\mathrm{ik}}}\prod_{i\notin z}{{\hat{q}}_{k}}^{c_{\mathrm{ik}}})}{\prod_{k}\prod_{i}{{\hat{p}}_{0k}}^{c_{\mathrm{ik}}}}$$

Where ${\hat{p}}_{0k}=\frac{C_{k}}{C}(={\hat{q}}_{0k})$ is the maximum likelihood estimator (MLE) of $p_{k}(=q_{k})$ under the null hypothesis. ${\hat{p}}_{k}$ and ${\hat{q}}_{k}$ are the MLEs of $p_{k}$ and $q_{k}$ under the alternative hypothesis.

The application of the Gini coefficient for the ordinal model was proposed by Kim and Jung (28). It assumes that if there is only one significant cluster $z^{*}$ . The x-coordinate of the point corresponding to the cluster on the Lorenz curve for the ordinal model is defined as


$$\frac{\sum_{k}k({\hat{p}}_{k}\sum_{k}\sum_{i\in z^{*}}c_{\mathrm{ik}})}{\sum_{k}kC_{k}}\;\;\;$$ (1)

and the y-coordinate is defined as


$$\frac{\sum_{k}\sum_{i\in z^{*}}c_{\mathrm{ik}}}{\sum_{k}C_{k}}\;\;\;$$ (2)

The Lorenz curve for the ordinal model is defined with the x-axis representing the cumulative proportion of weighted cases and the y-axis representing the cumulative proportion of total cases. Ordinal scores are assigned to ordered categories. The denominator is the weighted number of total cases. The numerator uses estimated number of cases derived from the total number of observations multiplied by the ML estimates of $p_{k}$ to avoid confusion from combined categories. Therefore, the point (*x*, *y*) represents that *y*(×100)% of total observations have *x*(×100)% of total attributes. When there were multiple significant clusters, we derived coordinates for each cluster by cumulating the numerators in Equations 1, 2. If there were J points ( $x_{j},y_{j}$ ), where j = 1, …, J, the Gini coefficient was computed as $\sum_{j=1}^{J+1}(x_{j-1}y_{j}-x_{j}y_{j-1})$ with $\left(x_{0},y_{0})=(0,0\right)$ and $\left(x_{J+1},y_{J+1})=(1,1\right)$ , according to Han et al. (27). Finally, we selected the MRCS with the highest Gini coefficient. Details of the Gini coefficient for the ordinal model are explained in the paper by Kim and Jung (28).

#### 2.2.2 Zero-inflated ordered logit (ZIOL) model

We suspect these zero fracture samples belong to one of two latent groups. The first group consists of genuine non-fractured individuals who are not susceptible to fractures, termed the excess group. The second group includes individuals who did not fracture during these five years but might have, had they lived in less walkable neighborhoods or engaged in less exercise. These individuals are called conditional zeros because their zero-fracture status is conditional on being susceptible to fractures. We suspect that these two types of zeros are driven by different patterns, hence, we choose ZIOL model to model the relationships. The ZIOL model comprises two processes: a binary logit model that determines whether an observation belongs to the excess group, and an ordered logit model that assesses the fracture-frequency level in the second group. For the cluster specific models, covariate selection was performed using a stepwise regression procedure based on the Akaike Information Criterion, implemented in R using the stepAIC function. The selection procedure was conducted separately for the high and low clusters, and therefore the covariates retained in the final models were not necessarily identical between the clusters.

ZIOL model was proposed by Kelley and Anderson (29). The calculation process is as follows. Let $y_{j}$ denote an ordinal measure representing the number of fractures experienced by older adult j during the five-year period from 2017 to 2021, with 6 levels ranging from 0 to 5. Let define the older adult j that is susceptible to fracture as $s_{j}$ = 1 and non-susceptible to fracture as $s_{j}$ = 0. With the logit model, the probability of susceptibility to fracture is given by


$$\mathrm{Pr}(s_{j}=1|z_{j})=F(z_{j}ϒ)$$

$z_{j}$ is a vector of covariates that determines susceptibility, ϒ is a vector of coefficients to be estimated, and F ( · ) is the logistic distribution function: $F(x)=\frac{e^{x}}{1+e^{x}}$ . Next, conditioning on $s_{j}$ = 1, fracture-frequency level $\tilde{y_{j}}$ is modeled using an ordered logit model, which may also include 0. The probabilities are given by


$$\mathrm{Pr}(\tilde{y_{j}}=h|,s_{j}=1|,x_{j})=F(k_{h}-x_{j}\beta )-F(k_{h-1}-x_{j}\beta )\;\;$$ (3)

Where $k_{-1}$ = −∞ , $k_{H}$ = +∞ , $x_{j}$ is a vector of covariates that determines frequency level (which can be the same as or different from $z_{j}$ ), β is a vector of coefficients and $k_{h}$ are boundary parameters that all have to be estimated. $s_{j}$ and $\tilde{y_{j}}$ are both unobservable in terms of the zeroes. The observable variable is $y_{j}=s_{j}\tilde{y_{j}}$ . Therefore, the zero outcome occurs when $s_{j}$ = 0 (the older adult is non-susceptible to fracture) or when $s_{j}$ = 1 and $\tilde{y_{j}}=0$ (the older adult is susceptible to fracture but experienced 0 fracture). To observe a positive $y_{j}$ , it needs both $s_{j}$ = 1 and $\tilde{y_{j}}>0$ . Thus, the distribution of $y_{j}$ can be expressed as follows


$$\mathrm{Pr}(y_{j})=\{\begin{array}{l} \mathrm{Pr}(y_{j}=0|z_{j},x_{j})\\ \mathrm{Pr}(y_{j}=h|z_{j},x_{j})\;h=1,2,3,4,5\; \end{array}$$

$$=\{\begin{array}{c} \mathrm{Pr}(s_{j}=0|z_{j})+\mathrm{Pr}(s_{j}=1|z_{j})\mathrm{Pr}({\tilde{y}}_{j}=0|s_{j}=1,x_{j})\\ \mathrm{Pr}(s_{j}=1|z_{j})\mathrm{Pr}({\tilde{y}}_{j}=h|s_{j}=1,x_{j})\;\;\;\;h=1,2,3,4,5 \end{array}\;\;$$ (4)

We can see that the probability of zero is inflated because it is the sum of the probability of being non-susceptible (from the logit model) and the probability of zero intensity (from the ordered logit model). Combining Equations 3, 4, the distribution of $y_{j}$ is as follows.


$$\mathrm{Pr}(y_{j})=\{\begin{array}{l} \mathrm{Pr}(y_{j}=0|z_{j},x_{j})\\ \mathrm{Pr}(y_{j}=h|z_{j},x_{j})\;\;\;\;\;\;\;\;h=1,2,3,4\\ \mathrm{Pr}(y_{j}=5|z_{j},x_{j}) \end{array}$$

$$=\{\begin{array}{l} \left\{1-F(z_{j}\gamma )\}-F(z_{j}\gamma )F(k_{0}-x_{j}\beta \right)\\ F(z_{j}\gamma )\{F(k_{h}-x_{j}\beta )-F(k_{h-1}-x_{j}\beta )\}\;\;\;\;\;\;\;\;h=1,2,3,4\\ F(z_{j}\gamma )\{1-F(k_{H-1}-x_{j}\beta )\} \end{array}$$

Using maximum likelihood criteria, the parameters can be estimated, with the following log-likelihood function


$$\ln L=\sum_{j=1}^{N}w_{j}\sum_{h=0}^{H}I(y_{j}=h)\ln\{\mathrm{Pr}(y_{j}=h|z_{j},x_{j})\}$$

Where N is total number of observations and H is the maximum fracture number, $w_{j}$ is an optional weight for each oversedation and


$$I(y_{j}=h)=\{\begin{array}{c} 1\;\text{if}\;y_{j}=h\\ 0\;\text{otherwise} \end{array}$$

Applying this method, we examined associations between each built environment attribute and fracture occurrence as well as fracture frequency within the high and low clusters separately.

## 3 Results

### 3.1 Cluster detection results

Using ordinal spatial scan statistics, we identified areas with high or low rates of larger number fracture cases in Kumamoto Prefecture. The Gini coefficient picked 20% as the optimal MRCS. The detected clusters are shown in Figure 2 and described in Table 1. Only two significant clusters were found: cluster 1, the most likely cluster, and cluster 2, the secondary cluster. Cluster 1 is the area with high rate of larger fracture numbers, while cluster 2 is the area with low rate of larger fracture numbers. Specifically, the most likely cluster is an area with rates for fractures numbering 0, 1, 2, and 3–5 in increasing sequence. Cluster 2 is an area with low rates of fractures numbering 3–5 compared to 1–2, which in turn have a lower rate than 0.

**Figure 2:**
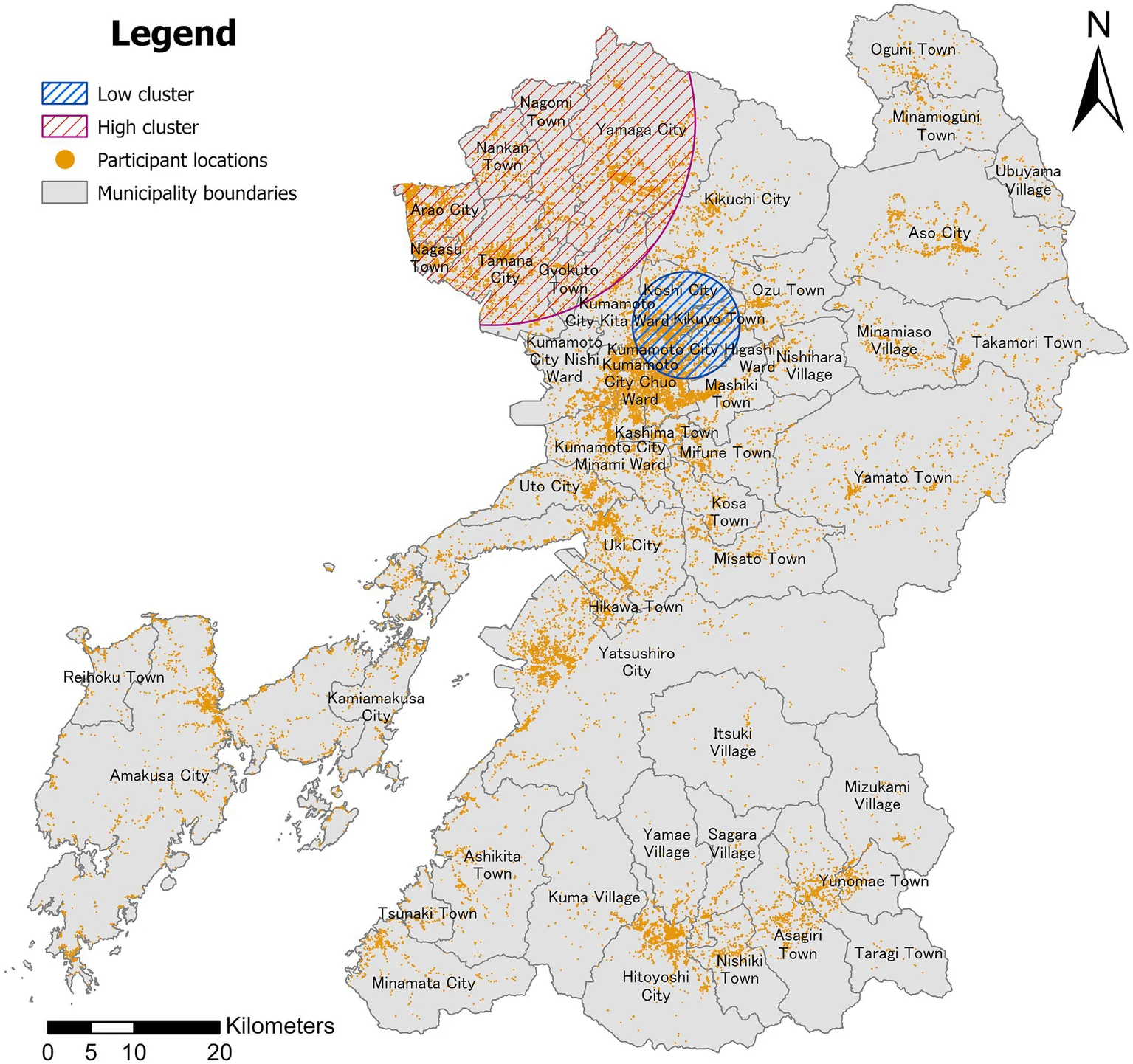
Spatial cluster analysis of fractures among older adults across Kumamoto Prefecture.

**Table 1:** Cluster detection analysis result for fractures among older adults in Kumamoto Prefecture using the ordinal scan statistic.

	Radius (km)	Population in area	Categories	#O/#E	RR	LRR	*p* value
High cluster	23.67	7,662	([0], [1], [2], [3, 4, 5])	(0.94, 1.18, 1.24, 1.39)	(0.93, 1.23, 1.32, 1.53)	70.78	< 0.001
Low cluster	6.22	3,789	([0], [1, 2], [3, 4, 5])	(1.05, 0.85, 0.60)	(1.06, 0.83, 0.57)	28.90	< 0.001

#O/^#^E, ratio of numbers observed versus expected; RR, relative risk; LRR, log-likelihood ratio.

### 3.2 Differences of characteristics between high and low clusters

Table 2 shows the personal characteristics and their differences between high and low clusters. In the low cluster area, 83.4% of older adults had experienced no fractures, whereas in the high cluster area, this proportion was only 74.3%. Except for Systolic Blood Pressure (SBP), Q3, and Q14, all other characteristics showed significant differences between high and low clusters. In the high cluster area, there were significantly higher average age, higher proportion of women, higher prevalence of estimated Glomerular Filtration Rate (eGFR) < 45 mL/min/1.73 m^2^, Hemoglobin (Hb) ≤ 11 g/dL, dementia, and musculoskeletal disorder, higher prevalence of using medications for hypertension, diabetes, dyslipidemia, and sleeping pills, as well as a higher number of medication types per month. Conversely, the high cluster area had a lower rate of HemoglobinA1c (HbA1c) ≥ 6.5% and smoking rate. Regarding lifestyle and self-perceived health status, individuals in the high cluster area reported poorer self-rated health and less satisfaction with daily life, poorer oral and cognitive function, lower exercise rate, less social engagement, and a higher rate of falls.

**Table 2:** Differences of personal characteristics between high and low clusters.

Variable	Total (*N* = 39,577)	High cluster (*N* = 7,662)	Low cluster (*N* = 3,789)	*p*-value
Fracture numbers, *n* (%)				< 0.001
0	31,328 (79.2)	5,693 (74.3)	3,158 (83.4)	
1	4,709 (11.9)	1,072 (14.0)	369 (9.7)	
2	1936 (4.9)	466 (6.1)	170 (4.5)	
3	813 (2.1)	224 (2.9)	43 (1.1)	
4	387 (1.0)	104 (1.4)	25 (0.7)	
5	404 (1.0)	103 (1.3)	24 (0.6)	
Gender, *n* (%)				< 0.001
Men	17,064 (43.1)	3,008 (39.3)	1772 (46.8)	
Women	22,513 (56.9)	4,654 (60.7)	2017 (53.2)	
Age (years), mean ± SD	80.8 (4.4)	81.7 (4.8)	79.9 (4.0)	< 0.001
BMI (kg/m^2^), mean ± SD	22.8 (3.3)	22.8 (3.3)	22.7 (3.1)	0.045
eGFR < 45 mL/min/1.73 m^2^, *n* (%)	4,410 (11.1)	959 (12.5)	295 (7.8)	< 0.001
Hb ≤ 11 g/dL, *n* (%)	1847 (4.7)	497 (6.5)	112 (3.0)	< 0.001
HbA1c ≥ 6.5%, *n* (%)	4,617 (11.7)	770 (10.0)	511 (13.5)	< 0.001
SBP ≥ 140 mmHg, *n* (%)	14,243 (36.0)	2,562 (33.4)	1,273 (33.6)	0.865
Dementia, *n* (%)	2,118 (5.4)	492 (6.4)	165 (4.4)	< 0.001
Musculoskeletal disorder, *n* (%)	30,497 (77.1)	6,063 (79.1)	2,719 (71.8)	< 0.001
Smoking status, *n* (%)				< 0.001
Yes	1,564 (4.0)	295 (3.9)	179 (4.7)	
Quitted	7,119 (18.0)	1,092 (14.3)	686 (18.1)	
No	30,894 (78.1)	6,275 (81.9)	2,924 (77.2)	
Q1: current health status, mean ± SD	3.6 (1.0)	3.6 (1.0)	3.7 (1.0)	< 0.001
Q2: satisfied with daily life, mean ± SD	3.4 (0.7)	3.4 (0.7)	3.5 (0.6)	< 0.001
Q3: eating three meals a day, *n* (%)	37,880 (95.7)	7,285 (95.1)	3,613 (95.4)	0.518
Q4: feeling more difficult in eating hard foods than six months ago, *n* (%)	10,131 (25.6)	2,314 (30.2)	797 (21.0)	< 0.001
Q5: sometimes choke on tea and soup, *n* (%)	7,333 (18.5)	1,594 (20.8)	670 (17.7)	< 0.001
Q6: having a weight loss of more than 2 kg in 6 months, *n* (%)	4,698 (11.9)	966 (12.6)	386 (10.2)	< 0.001
Q7: walking speed became slower, *n* (%)	21,801 (55.1)	4,627 (60.4)	1864 (49.2)	< 0.001
Q8: had falls in the past year, *n* (%)	7,135 (18.0)	1,632 (21.3)	583 (15.4)	< 0.001
Q9: doing exercises such as walking at least once a week, *n* (%)	24,299 (61.4)	4,315 (56.3)	2,681 (70.8)	< 0.001
Q10: having been told by others that you are forgetful, *n* (%)	5,539 (14.0)	1,215 (15.9)	440 (11.6)	< 0.001
Q11: sometimes forgot today’s date, *n* (%)	8,900 (22.5)	1995 (26.0)	805 (21.2)	< 0.001
Q12: going out at least once a week, *n* (%)	35,789 (90.4)	6,774 (88.4)	3,503 (92.5)	< 0.001
Q13: having regular interactions with family and friends, *n* (%)	37,688 (95.2)	7,251 (94.6)	3,593 (94.8)	< 0.001
Q14: having someone nearby to consult with when feeling unwell, *n* (%)	38,019 (96.1)	7,326 (95.6)	3,653 (96.4)	0.667
Hypertension medication, *n* (%)	25,118 (63.5)	5,159 (67.3)	2064 (54.5)	< 0.001
Diabetes medication, *n* (%)	4,980 (12.6)	998 (13.0)	414 (10.9)	0.001
Dyslipidemia medication, *n* (%)	15,786 (39.9)	3,363 (43.9)	1,320 (34.8)	< 0.001
Sleeping pill, *n* (%)	10,266 (25.9)	2039 (26.6)	925 (24.4)	0.011
Number of medication types per month, mean ± SD	4.0 (3.1)	4.3 (3.1)	3.3 (2.8)	< 0.001

BMI, Body Mass Index; SBP, Systolic Blood Pressure; HbA1c, HemoglobinA1c; eGFR, estimated Glomerular Filtration Rate; Hb, Hemoglobin; SD, Standard Deviation.

Data are shown as the mean (SD) or number (percentage).

The *p*-value indicates the significance of the comparison between high cluster samples and low cluster samples.

Table 3 shows the characteristics of built environment attributes and their differences between high and low clusters. Significant differences were observed in all six attributes between high and low clusters. High cluster samples were residing in neighborhoods with higher average population density, intersection density, walk score, mix of function, accessibility of bus services and lower average slope. Specifically, the average population density in the low cluster was over three times that of the high cluster, and intersection density was more than twice as high. The walk score and mix of function in the low cluster were nearly double compared to the high cluster. Additionally, accessibility of bus services in the low cluster was more than one and a half times that in the high cluster.

**Table 3:** Differences of built environment attributes between high and low clusters.

Variable	Range	High cluster (*N* = 7,662)	Low cluster (*N* = 3,789)	*p*-value
Walk score	0–100	15.9 (19.3)	27.5 (19.5)	< 0.001
Mix of function	0–100	32.0 (32.6)	60.7 (26.0)	< 0.001
Population density^a^	1–1,188	126.4 (116.8)	408.4 (196.5)	< 0.001
Intersection density^b^	0–211	37.8 (31.5)	97.3 (45.3)	< 0.001
Accessibility of bus services	0–100	30.4 (32.0)	49.3 (32.2)	< 0.001
Slope^c^	0–25	3.1 (3.3)	2.2 (2.9)	< 0.001

Data are shown as the mean ± SD.

^a^Numbers of persons at each 5th mesh (length side ≈ 250 m) where each older adult resided.

^b^Numbers of intersections at each 500 m network distance buffer zone from each older adult resided.

^c^Slope exceeding 25° was considered an outlier and capped at 25° (outlier samples = 14).

The *p*-value indicates the significance of the comparison between high cluster samples and low cluster samples.

### 3.3 Zero-inflated ordered logit model result for each cluster

Table 4 shows the results of ZIOL model for the low cluster. We modeled fracture frequency levels for samples who were susceptible to fracture as a function of age, gender, BMI, dementia, musculoskeletal disorder, Q6, Q7, Q8, Q10, Q11, hypertension medication and each of the built environment attributes. The occurrence was modeled as a function of age, gender, BMI, dementia, musculoskeletal disorder, Q7, Q8, Q10, Q11 and each of the built environment attributes. The covariates adjusted for each process were also listed below the table. Both the ordered logit process and logit process did not reveal any significant relationships between fracture frequency or occurrence and built environment attributes.

**Table 4:** Zero-inflated ordered logit model result for the low cluster.

Variable^a^	Logit process^b^		Ordered logit process^c^	
	OR	95% CI	OR	95% CI
Walk score	1.35	(0.60, 3.03)	1.25	(0.50, 3.10)
Mix of function	0.90	(0.44, 1.85)	1.44	(0.70, 2.92)
Population density	1.27	(0.36, 4.53)	1.38	(0.21, 9.14)
Intersection density	0.77	(0.24, 2.51)	3.38	(0.72, 16.02)
Accessibility of bus services	1.37	(0.80, 2.32)	1.02	(0.59, 1.76)
Slope	1.14	(0.28, 4.67)	0.43	(0.10, 1.94)

^a^Each built environment attribute was included separately to examine its individual association with fracture occurrence.

^b^Adjustment for age, gender, BMI, dementia, musculoskeletal disorder, Q7, Q8, Q10, Q11.

^c^Adjustment for age, gender, BMI, dementia, musculoskeletal disorder, Q6, Q7, Q8, Q10, Q11, hypertension medication.

Table 5 shows the results of ZIOL model for the high cluster. We modeled fracture frequency levels for samples who were susceptible to fracture as a function of age, gender, eGFR, Hb, dementia, musculoskeletal disorder, Q2, Q6, Q8, Q11, hypertension medication, dyslipidemia medication, number of medication types per month and each of the built environment attributes. The occurrence was modeled as a function of age, gender, eGFR, Hb, dementia, musculoskeletal disorder, Q2, Q4, Q6, Q8, Q11, dyslipidemia medication and each of the built environment attributes. The covariates adjusted for each process were listed below the table. The ordered logit process did not show any significant relationships between fracture frequency and built environment attributes in the susceptible group. On the contrary, in the logit process, four individual built environment attributes showed statistically significant associations with fracture occurrence in their respective models. Specifically, the highest population density lowered the odds by a factor of 0.50 (50% decrease in odds) compared to the lowest population density (*p* < 0.001), which was the largest reduction observed. The highest intersection density lowered the odds by a factor of 0.64 (36% decrease in odds) compared to the lowest intersection density (*p* < 0.01). The highest accessibility of bus services significantly lowered the odds by a factor of 0.75 (25% decrease in odds) (*p* < 0.01). Similarly, the highest walk score marginally significantly lowered the odds of being susceptible to fracture by a factor of 0.75 compared to the lowest walk score. Finally, the highest mix of function lowered the odds by a factor of 0.82 (18% decrease in odds) compared to the lowest mix of function (*p* < 0.05). Nevertheless, no significant relationship was observed between slope and occurrence of fracture.

**Table 5:** Zero-inflated ordered logit model result for the high cluster.

Variable^a^	Logit process^b^		Ordered logit process^c^	
	OR	95% CI	OR	95% CI
Walk score	0.75^†^	(0.55, 1.01)	0.93	(0.56, 1.54)
Mix of function	0.82^*^	(0.68, 0.98)	0.93	(0.69, 1.25)
Population density	0.50^***^	(0.35, 0.71)	0.89	(0.49, 1.62)
Intersection density	0.64^**^	(0.46, 0.89)	1.14	(0.67, 1.95)
Accessibility of bus services	0.75^**^	(0.63, 0.91)	1.22	(0.92, 1.62)
Slope	1.12	(0.71, 1.77)	1.79	(0.87, 3.68)

^a^Each built environment attribute was included separately to examine its individual association with fracture occurrence.

^b^Adjustment for age, gender, eGFR, Hb, dementia, musculoskeletal disorder, Q2, Q4, Q6, Q8, Q11, dyslipidemia medication.

^c^Adjustment for age, gender, eGFR, Hb, dementia, musculoskeletal disorder, Q2, Q6, Q8, Q11, hypertension medication, dyslipidemia medication, number of medication types per month.

^†^Significance at *p* < 0.10. *Significance at *p* < 0.05. **Significance at *p* < 0.01. ***Significance at *p* < 0.001.

Figure 3 illustrates the relationship between each fracture outcome and each level of the built environment attributes. The vertical axis represents the expected probabilities of each fracture outcome, while the horizontal axis represents the levels of each built environment attribute. The probability of 0 fracture consistently increased with higher levels of walk score, mix of function, population density, intersection density, and accessibility of bus services, reaching its peak at the highest levels. Conversely, the probability of 1 fracture consistently decreased as the levels of these five built environment attributes increased. Notably, the most pronounced changes in probabilities were observed for population density, followed by intersection density. The probability of 2 fractures also consistently decreased as the levels of these five built environment attributes increased, though the magnitudes of these decreases were comparatively small. However, the probability of more than 2 fractures remained almost unchanged as the levels of these five built environment attributes increased, except for a slight decrease in the probability of 3 fractures with increasing population density. The probability of 0 or 1 fracture decreased with higher slope, while probability of 2 or more than 2 fractures increased with higher slope.

**Figure 3:**
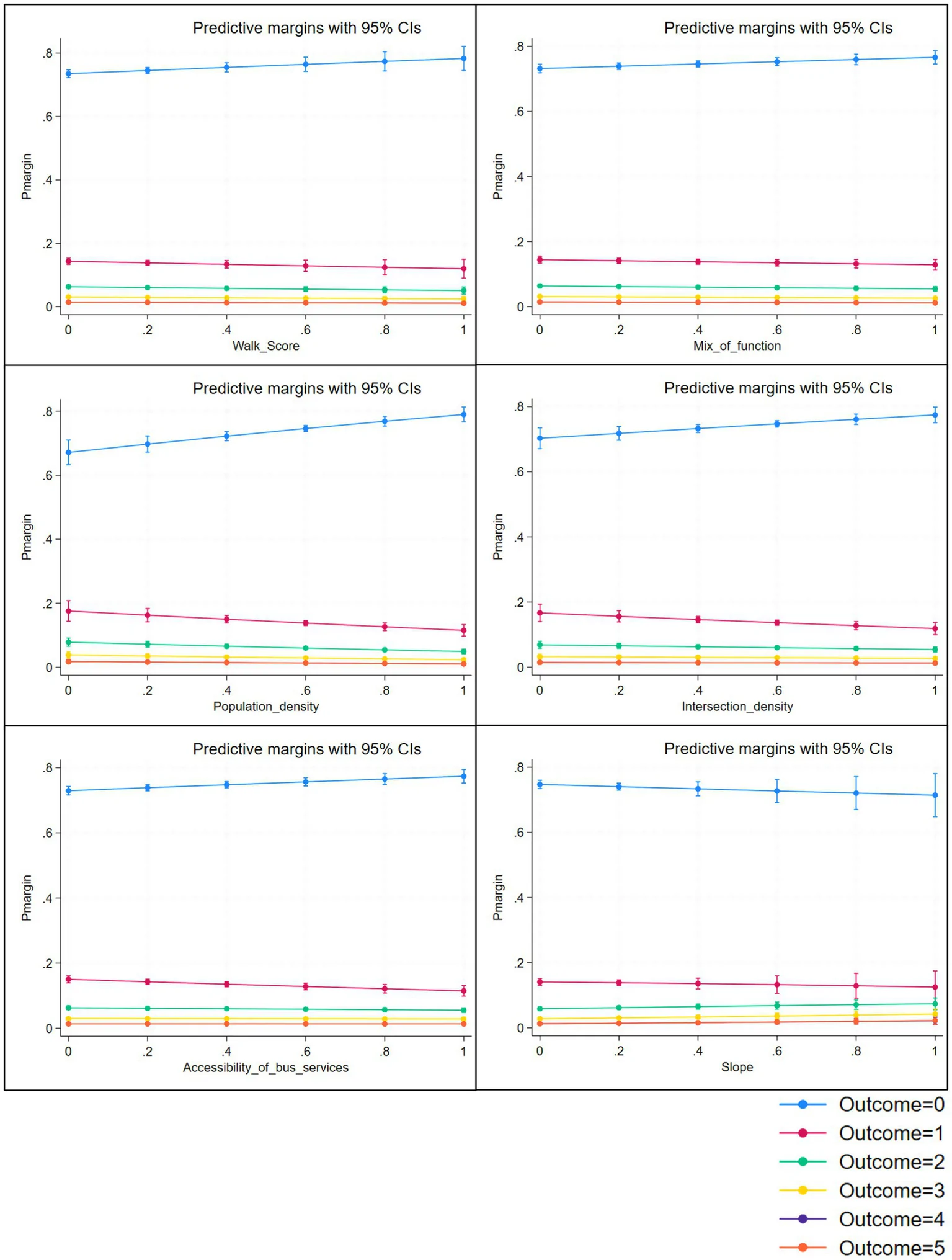
Marginal effects of each built environment attribute on each fracture outcome probability.

Figure 4 shows the relationship between susceptibility to fracture and each level of the built environment attributes. Nearly 29% of respondents were susceptible to fractures when the walk score, mix of function, or accessibility of bus services was zero, with the probability decreasing to below 25% for walk score and accessibility of bus services and to nearly 26% for mix of function as these attributes increased to the highest. More than 35% of respondents were susceptible to fractures when population density was zero, with the probability decreasing to below 25% as population density increased to the highest. Similarly, nearly 32% of respondents were susceptible to fractures when intersection density was zero, with the probability decreasing to below 25% as intersection density increased to the highest. For slope, less than 30% of respondents were susceptible to fractures when there was no slope, but the probability increased to around 31% with the highest slope.

**Figure 4:**
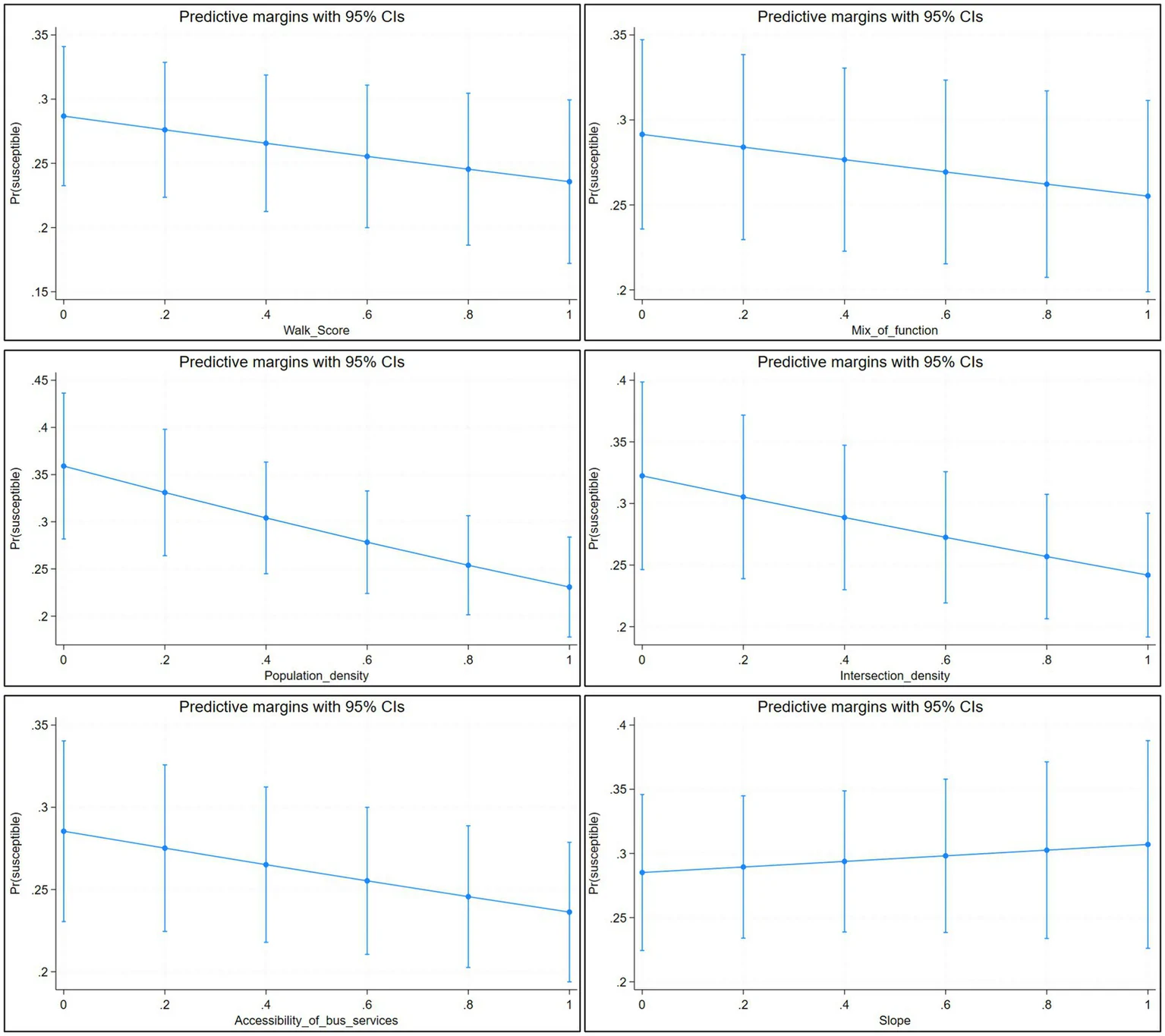
Marginal effects of each built environment attribute on the probability of susceptibility to fracture.

## 4 Discussion

Using ordinal scan statistics with fine-grained residential point data, we detected one high and one low spatial clusters of fractures among older adults in Kumamoto Prefecture. Then, based on ZIOL model, we found that none of the built environment attributes was linked to either the occurrence or the frequency of fracture in the low cluster areas. In the high cluster areas, four individual built environment attributes—population density, intersection density, accessibility of bus services, and mix of function—showed statistically significant associations with fracture occurrence in their respective models, while none of the built environment attributes showed a statistically significant association with fracture frequency. The walk score showed a marginally significant association with fracture occurrence in the high cluster areas.

Older adults are at high risk of fracture due to low bone mineral density (30). Identifying fracture hotspots among older adults and understanding the associated factors is of great significance. Although previous studies have investigated fracture rates in Japan, especially hip fractures, most have focused on national descriptive trends (31–33). One study investigated the geographical variation of fracture in Japan and found that there were east–west regional differences in the standardized incidence ratio of hip fractures for all genders, with the highest incidence in Minami Kyushu (34). However, this study used the prefecture as the study area. To the best of our knowledge, this is the first study to analyze the spatial pattern of fractures using fine-grained residential point data. Point data is rarely used in epidemiological analyses because aggregated health data is more readily accessible. Most studies utilize larger geographic units, such as census tracts, towns, or cities, for their analyses. For those using point data, a review revealed that over half employed spatial scan statistics to analyze spatial patterns of address location data, with over 80% using the Bernoulli model for case–control data (18). In our study, using a five-year period of fracture data, we conducted spatial scan statistics with an ordinal model to detect fracture hotspots. This approach not only identifies accurate hotspots but also provides the relative risk for each category or combined categories. Applying this method, Fuchs et al. analyzed the spatial pattern of damage to buildings located in settlements affected by torrential hazards in five study sites (23). Similarly, Sulistyawati et al. detected measles hotspots using ordinal scan statistic model (21). Notably, these studies analyzed a limited number of cases, with no more than 50 cases in each study site. In contrast, our study geocoded 39,577 participants across the entire prefecture with residential locations and detected the clusters with those fine-grained residential point data. We found that the high cluster area was in the northwest part of the prefecture, name Tamana area and Yamaga city. This area is characterized by an older average age, a high proportion of women, poorer physical and cognitive function, as well as lower rates of outing and exercise. The low cluster was in the northeast direction of Kumamoto city, including Koshi city and Kikuyo town. The older adults in this area have a lower average age and better health conditions. Although the proportion of women is still higher than that of men, it is much lower than the average.

Our findings revealed different patterns of association between built environment attributes and fracture outcomes across the identified spatial clusters. Specifically, significant associations were observed only in the high cluster areas, where four individual built environment attributes showed statistically significant associations with fracture occurrence in their respective models. In contrast, no significant relationships were identified in the low cluster areas, suggesting that the built environment’s association with fracture risk may be dependent on the context. Only three previous studies have examined the relationship between walkability and fracture outcomes. A Chinese cohort study indicated that every IQR increment in neighborhood walkability was associated with a hazard ratio of 0.88 (95% CI = 0.83–0.92) among people aged 40 years or older (10). Similarly, a Japanese study identified that mix of function was a protective factor for older adults’ fracture health (OR = 0.92, 95%CI = 0.85–0.99), walk score and accessibility of bus services were marginally significantly associated with reduced fracture risk among older adults aged 75 years or older (11). In addition, a US study systematically examined the relationship between fracture and walkability among women ages 50–79 years using a nationally representative sample. This study found a non-liner relationship: high walkability was related to decreased risk (hazard ratio = 0.98), whereas intermediate walkability was related to an increased risk for total fractures compared to low walkability (hazard ratio = 1.04). Moreover, urban–rural differences also existed in the association between walkability and total fractures, suggesting that walkability measures developed for urban areas may not fully capture features that promote walking in rural environments (8). In our study, we observed several statistically significant attribute-specific associations with fracture occurrence in the high cluster areas. The built environment attributes included population density, intersection density, accessibility of bus services, and mix of function. Among these, population density demonstrated the most significant association with fracture occurrence (OR = 0.50, 95% CI = 0.35–0.71), indicating that older adults residing in the highest population density areas were only half as likely to experience fractures compared to those in the lowest density areas. This finding underscores the potential role of densely populated environment, which may offer greater access to healthcare services, improved infrastructure for pedestrian mobility, and increased social interactions that encourage physical activity and community cohesion. However, these mechanisms were not directly examined in this study. Following population density, intersection density also exhibited significant association (OR = 0.64, 95% CI = 0.46–0.89). This finding indicates that older adults living in areas with higher street connectivity had a lower risk of experiencing fractures. The presence of more intersections and well-connected street networks may facilitate shorter and safer walking routes, increased pedestrian activity, and improved accessibility to daily services, thereby contributing to enhanced mobility and fall prevention. While the built environment attributes were significantly associated with fracture occurrence in high cluster areas, no significant association was observed between these attributes and fracture frequency. This distinction suggests that while walkable environment may effectively reduce the probability of fracture occurrence, they do not necessarily mitigate the frequency of fractures once they occur. One potential explanation for this finding is that individual-level characteristics may overshadow the association of walkability with fracture frequency. Key factors such as bone density, frailty, pre-existing musculoskeletal disorder may play a more important role in influencing the frequency of fractures than external environment. These findings suggest that while built environment plays a critical role in fracture prevention, additional interventions targeting individual health conditions and fall prevention programs are essential to reduce the frequency of fractures among older adults. Different with high cluster areas, we did not observe any significant associations between built environment attributes and fracture outcomes in the low cluster areas. Several factors may explain the absence of association. First, older adults residing in low cluster areas tended to have better overall health status, which could have contributed to a lower baseline risk of fractures. Specifically, these individuals exhibited better physical performance, oral function, lower fall incidence, and fewer musculoskeletal disorders, all of which are known to reduce fracture susceptibility. Second, the overall fracture incidence in low cluster areas was 9.1% lower than in high cluster areas, which may have contributed to reduced statistical power in detecting significant relationships. A lower incidence of fractures in these areas suggests that fracture occurrence may be associated with factors other than the built environment, such as personal health status, lifestyle behaviors, and socioeconomic conditions. Overall, our study provides important insights into the role of built environment attributes, particularly population density and intersection density in the high cluster areas, in mitigating fracture susceptibility among older adults. These findings can inform future WHO initiatives aimed at promoting independent aging by emphasizing the importance of urban planning strategies that enhance density and connectivity. Additionally, our findings pinpoint regions with the highest fracture risk, offering a foundation for targeted interventions and preventive measures by local governments. Conversely, in the low cluster areas, public health initiatives may need to prioritize individual-level interventions, such as bone health screenings, strength training programs, and nutrition interventions, to further mitigate fracture risk.

This study has some limitations. Firstly, out of the 39,577 samples, 590 (1.49%) samples could only be geocoded at the block level. However, since this had almost no impact on the analysis and results, we have included these samples in all analyses. Secondly, due to data limitations, we used only a five-year period for fracture data and had cross-sectional data for other variables, which precluded the establishment of a longitudinal study design. Thirdly, aggregating annual fracture records into the cumulative number of fracture frequence over five years may have resulted in the loss of temporal information, such as the timing and sequence of fracture events. Future studies using longitudinal approaches are needed to better examine temporal patterns of fracture occurrence. Fourthly, each built environment attribute was modeled separately, and the covariate selection procedure was conducted independently within the high and low clusters. Therefore, the estimated associations cannot be interpreted as mutually adjusted independent effects, and direct comparison of coefficients across clusters is limited. Formal interaction models using a common *a priori* covariate set would be useful in future research to test whether associations differ statistically between spatial contexts. Additionally, built environment characteristics were measured at baseline and may not reflect changes in neighborhood environments during follow-up. Residential relocation after fracture events may also have introduced potential exposure misclassification. Future studies with repeated exposure measurements and time-varying data are needed to further examine these temporal relationships.

Despite the limitations, this study has some strengths. Firstly, we utilized fine-grained residential point data for cluster detection, which allowed for precise hotspot detection and a better understanding of the characteristics within hotspot areas. This approach provides a detailed spatial characterization of fracture-frequency patterns and assists local governments in implementing urgent interventions and preventive strategies in high risk areas, thereby promoting active and independent aging. Secondly, we employed an ordinal model for cluster detection using five years of fracture data, which is distinct from case–control studies that rely on only one year of fracture records. This method preserves the ordinal information in the fracture-frequency outcome and ensures that the identified clusters accurately reflect regions with severe fracture health issues. Thirdly, we performed a zero-inflated ordered logit model instead of generalized ordered logit model, enabling a separate examination of fracture occurrence and frequency in relation to each built environment attribute. Our findings suggest that built environment attributes showed associations with the occurrence related logit process in the high cluster, while no statistically significant associations with fracture frequency were observed in the ordered logit process. Future study should explore the mechanisms underlying the observed associations between neighborhood built environment and fracture outcomes.

## 5 Conclusion

Using ordinal scan statistics with a five-year period of fracture data, we identified one high and one low fracture clusters among Japanese older adults. We then applied a zero-inflated ordered logit (ZIOL) model to each cluster to examine associations between built environment attributes and fracture outcomes. No significant associations between built environment attributes and fracture occurrence or fracture frequency were observed in the low fracture cluster. In the high fracture cluster, four individual built environment attributes were significantly associated with lower odds of fracture occurrence in their respective models, with population density being particularly significant. This suggests that while built environment attributes may not necessarily be associated with reduced fracture frequency for susceptible group, whereas they are related to lower risk of experiencing fracture for non-susceptible group. These findings provide valuable insights that can inform initiatives aimed at encouraging active and independent aging.

## Data Availability

The data analyzed in this study is subject to the following licenses/restrictions: Health data were obtained from the Kumamoto Prefecture Senior Citizens’ Medical and Wide Area Alliance. The authors do not have permission to share data. Requests to access these datasets should be directed to Kumamoto Prefecture Senior Citizens’ Medical and Wide Area Alliance through the website of https://www.kumamoto-kouikirengo.jp/.

## References

[1] Cummings SR Melton LJ. Epidemiology and outcomes of osteoporotic fractures. Lancet. (2002) 359:1761–7. doi: 10.1016/S0140-6736(02)08657-9 12049882

[2] Odén A McCloskey EV Kanis JA Harvey NC Johansson H. Burden of high fracture probability worldwide: secular increases 2010–2040. Osteoporos Int. (2015) 26:2243–8. doi: 10.1007/s00198-015-3154-6, 26018089

[3] Cooper C Campion G Melton LJ. Hip fractures in the elderly: a world-wide projection. Osteoporos Int. (1992) 2:285–9. doi: 10.1007/BF01623184, 1421796

[4] Japanese Ministry of Health, Labor and Welfare (2023). National Medical Expenditure Survey Tokyo, Japan: Ministry of Health, Labour and Welfare. Available online at: https://www.mhlw.go.jp/toukei/saikin/hw/k-iryohi/21/dl/data.pdf (Accessed September 23, 2026).

[5] National Institute of Population and Social Security Research (2017). Population Projections for Japan (2017):2016 to 2065 Tokyo, Japan: National Institute of Population and Social Security Research. Available online at: https://www.ipss.go.jp/pp-zenkoku/e/zenkoku_e2017/pp29_summary.pdf

[6] Ström O Borgström F Kanis JA Compston J Cooper C McCloskey EV . Osteoporosis: burden, health care provision and opportunities in the EU. Arch Osteoporos. (2011) 6:59–155. doi: 10.1007/s11657-011-0060-1 22886101

[7] Michael YL Yen IH. Aging and place - Neighborhoods and health in a world growing older. J Aging Health. (2014) 26:1251–60. doi: 10.1177/0898264314562148, 25502240 PMC10339444

[8] Wende ME. Neighborhood socioeconomic status, walkability, and green space and risk for falls and fractures among postmenopausal women: the women’s health initiative. Diss Abstr Int Sect B Sci Eng. University of South Carolina. (2021) 83. Available online at: https://scholarcommons.sc.edu/etd/6480

[9] Costa AG Wyman A Siris ES Watts NB Silverman S Saag KG . When, where and how osteoporosis-associated fractures occur: an analysis from the global longitudinal study of osteoporosis in women (GLOW). PLoS One. (2013) 8:e83306. doi: 10.1371/journal.pone.0083306, 24349484 PMC3859637

[10] Zhu Z Yang Z Xu L Wu Y Yu L Shen P . Exposure to Neighborhood walkability and residential greenness and incident fracture. JAMA Netw Open. (2023) 6:e2335154. doi: 10.1001/jamanetworkopen.2023.35154, 37768665 PMC10539990

[11] Zhou Q Homma R Ni Lwin ZN. Exploring the relationship between neighborhood walkability and fracture risk among older people in Japan. J Transp Health. (2024) 38:101845. doi: 10.1016/j.jth.2024.101845

[12] Yiannakoulias N Rowe BH Svenson LW Schopflocher DP Kelly K Voaklander DC. Zones of prevention: the geography of fall injuries in the elderly. Soc Sci Med. (2003) 57:2065–73. doi: 10.1016/S0277-9536(03)00081-9, 14512238

[13] Macintyre S Maciver S Sooman A. Area, class and health: should we be focusing on places or people? J Soc Policy. (1993) 22:213–34. doi: 10.1017/S0047279400019310

[14] Sadaqa M Németh Z Makai A Prémusz V Hock M. Effectiveness of exercise interventions on fall prevention in ambulatory community-dwelling older adults: a systematic review with narrative synthesis. Front Public Heal. (2023) 11:1–14. doi: 10.3389/fpubh.2023.1209319, 37601180 PMC10435089

[15] Ministry of Land, Infrastructure, Transport and Tourism (2014). Handbook for Urban Structure Evaluation Tokyo, Japan: Ministry of Land, Infrastructure, Transport and Tourism. Available online at: https://www.mlit.go.jp/common/001104012.pdf (Accessed September 23, 2026).

[16] Mclafferty S. Disease cluster detection methods: recent developments and public health implications. Ann GIS. (2015) 21:127–33. doi: 10.1080/19475683.2015.1008572

[17] Kulldorff M. A spatial scan statistic. Commun Stat. (1997) 26:1481–96. doi: 10.1080/03610929708831995

[18] Fritz CE Schuurman N Robertson C Lear S. A scoping review of spatial cluster analysis techniques for point-event data. Geospat Health. (2013) 7:183. doi: 10.4081/gh.2013.79, 23733283

[19] Amin R Hendryx M Shull M Bohnert A. A cluster analysis of pediatric cancer incidence rates in Florida: 2000–2010. Stat Public Policy. (2014) 1:69–77. doi: 10.1080/2330443X.2014.928245

[20] Bell N Kruse S Simons RK Brussoni M. A spatial analysis of functional outcomes and quality of life outcomes after pediatric injury. Inj Epidemiol. (2014) 1:16. doi: 10.1186/s40621-014-0016-1, 26613070 PMC4648946

[21] Sulistyawati S Sumiana S. Measles cluster detection using ordinal scan statistic model. Mater Sociomed. (2018) 30:282. doi: 10.5455/msm.2018.30.282-286, 30936793 PMC6377924

[22] Westercamp N Moses S Agot K Ndinya-Achola JO Parker C Amolloh KO . Spatial distribution and cluster analysis of sexual risk behaviors reported by young men in Kisumu, Kenya. Int J Health Geogr. London, UK: BioMed Central. (2010) 9:24. doi: 10.1186/1476-072X-9-24, 20492703 PMC2881901

[23] Fuchs S Ornetsmüller C Totschnig R. Spatial scan statistics in vulnerability assessment: an application to mountain hazards. Nat Hazards. (2012) 64:2129–51. doi: 10.1007/s11069-011-0081-5

[24] Amin RW Kocak S Sevil HE Peterson GP Hamilton JT Rivera B . A spatio-temporal cluster analysis of structurally deficient bridges in the contiguous USA. Dev Built Environ. (2020) 4:100034. doi: 10.1016/j.dibe.2020.100034, 42574925

[25] Jung I Kulldorff M Klassen AC. A spatial scan statistic for ordinal data. Stat Med. (2007) 26:1594–607. doi: 10.1002/sim.2607, 16795130

[26] Kulldorff M. (2022). SaTScan User Guide 10.1 Available online at: https://www.satscan.org (Accessed September 23, 2026).

[27] Han J Zhu L Kulldorff M Hostovich S Stinchcomb DG Tatalovich Z . Using Gini coefficient to determining optimal cluster reporting sizes for spatial scan statistics. Int J Health Geogr. (2016) 15:27. doi: 10.1186/s12942-016-0056-6, 27488416 PMC4971627

[28] Kim S Jung I. Optimizing the maximum reported cluster size in the spatial scan statistic for ordinal data. PLoS One. (2017) 12:e0182234. doi: 10.1371/journal.pone.0182234, 28753674 PMC5533428

[29] Kelley ME Anderson SJ. Zero inflation in ordinal data: incorporating susceptibility to response through the use of a mixture model. Stat Med. (2008) 27:3674–88. doi: 10.1002/sim.3267, 18351711 PMC2572084

[30] Styrkarsdottir U Halldorsson BV Gretarsdottir S Gudbjartsson DF Walters GB Ingvarsson T . Multiple genetic loci for bone mineral density and fractures. N Engl J Med. (2008) 358:2355–65. doi: 10.1056/nejmoa0801197, 18445777

[31] Hagino H Endo N Harada A Iwamoto J Mashiba T Mori S . Survey of hip fractures in Japan: recent trends in prevalence and treatment. J Orthop Sci. (2017) 22:909–14. doi: 10.1016/j.jos.2017.06.003, 28728988

[32] Orimo H Yaegashi Y Hosoi T Fukushima Y Onoda T Hashimoto T . Hip fracture incidence in Japan: estimates of new patients in 2012 and 25-year trends. Osteoporos Int. (2016) 27:1777–84. doi: 10.1007/s00198-015-3464-8, 26733376 PMC4873530

[33] Takusari E Sakata K Hashimoto T Fukushima Y Nakamura T Orimo H. Trends in hip fracture incidence in Japan: estimates based on Nationwide hip fracture surveys from 1992 to 2017. JBMR Plus. (2021) 5:e10428. doi: 10.1002/jbm4.10428, 33615101 PMC7872333

[34] Orimo H Yaegashi Y Onoda T Fukushima Y Hosoi T Sakata K. Hip fracture incidence in Japan: estimates of new patients in 2007 and 20-year trends. Arch Osteoporos. (2009) 4:71–7. doi: 10.1007/s11657-009-0031-y, 20234789 PMC2836738

