# Supplementary material for: Neighborhood built environment and fracture risk among older adults: comparing high versus low clusters
Source: Front Public Health. 2026 Oct 16;14:1947343. doi: 10.3389/fpubh.2026.1947343 (PMC13719309; doi:10.3389/fpubh.2026.1947343)
Supplement: Supplementary file 1 [file Table_1.DOCX]

**Table S1.** Zero-inflated ordered logit model result for the low cluster

| Variable ^a^ | Logit process ^b^ | | | | Ordered logit process ^c^ | | | |
| --- | --- | --- | --- | --- | --- | --- | --- | --- |
|  | OR | Std. err. | P>\|z\| | 95% CI | OR | Std. err. | P>\|z\| | 95% CI |
| Walk Score | 1.35 | 0.56 | 0.471 | (0.60, 3.03) | 1.25 | 0.58 | 0.628 | (0.50, 3.10) |
| Mix of function | 0.90 | 0.33 | 0.774 | (0.44, 1.85) | 1.44 | 0.52 | 0.320 | (0.70, 2.92) |
| Population density | 1.27 | 0.82 | 0.711 | (0.36, 4.53) | 1.38 | 1.33 | 0.737 | (0.21, 9.14) |
| Intersection density | 0.77 | 0.46 | 0.667 | (0.24, 2.51) | 3.38 | 2.68 | 0.124 | (0.72, 16.02) |
| Accessibility of bus services | 1.37 | 0.37 | 0.248 | (0.80, 2.32) | 1.02 | 0.28 | 0.938 | (0.59, 1.76) |
| Slope | 1.14 | 0.82 | 0.853 | (0.28, 4.67) | 0.43 | 0.33 | 0.274 | (0.10, 1.94) |

^a^ Each built environment attribute was included separately to examine its individual association with fracture occurrence.

^b^ Adjustment for age, gender, BMI, dementia, musculoskeletal disorder, Q7, Q8, Q10, Q11.

^c^ Adjustment for age, gender, BMI, dementia, musculoskeletal disorder, Q6, Q7, Q8, Q10, Q11, hypertension medication.

**Table S2.** Zero-inflated ordered logit model result for the high cluster

| Variable ^a^ | Logit process ^b^ | | | | Ordered logit process ^c^ | | | |
| --- | --- | --- | --- | --- | --- | --- | --- | --- |
|  | OR | Std. err. | P>\|z\| | 95% CI | OR | Std. err. | P>\|z\| | 95% CI |
| Walk Score | 0.75† | 0.11 | 0.055 | (0.55, 1.01) | 0.93 | 0.24 | 0.781 | (0.56, 1.54) |
| Mix of function | 0.82* | 0.07 | 0.025 | (0.68, 0.98) | 0.93 | 0.14 | 0.630 | (0.69, 1.25) |
| Population density | 0.50*** | 0.09 | 0.000 | (0.35, 0.71) | 0.89 | 0.27 | 0.710 | (0.49, 1.62) |
| Intersection density | 0.64** | 0.11 | 0.008 | (0.46, 0.89) | 1.14 | 0.31 | 0.622 | (0.67, 1.95) |
| Accessibility of bus services | 0.75** | 0.07 | 0.003 | (0.63, 0.91) | 1.22 | 0.18 | 0.171 | (0.92, 1.62) |
| Slope | 1.12 | 0.26 | 0.619 | (0.71, 1.77) | 1.79 | 0.66 | 0.115 | (0.87, 3.68) |

^a^ Each built environment attribute was included separately to examine its individual association with fracture occurrence.

^b^ Adjustment for age, gender, eGFR, Hb, dementia, musculoskeletal disorder, Q2, Q4, Q6, Q8, Q11, dyslipidemia medication.

^c^ Adjustment for age, gender, eGFR, Hb, dementia, musculoskeletal disorder, Q2, Q6, Q8, Q11, hypertension medication, dyslipidemia medication, number of medication types per month.

†Significance at P < 0.10. *Significance at P < 0.05. **Significance at P < 0.01. ***Significance at P < 0.001.
